# Partner Choice Drives the Evolution of Cooperation via Indirect Reciprocity

**DOI:** 10.1371/journal.pone.0129442

**Published:** 2015-06-09

**Authors:** Gilbert Roberts

**Affiliations:** Centre for Behaviour and Evolution, Institute of Neuroscience, Newcastle University, Newcastle upon Tyne, United Kingdom

## Abstract

Indirect reciprocity potentially provides an important means for generating cooperation based on helping those who help others. However, the use of ‘image scores’ to summarize individuals’ past behaviour presents a dilemma: individuals withholding help from those of low image score harm their own reputation, yet giving to defectors erodes cooperation. Explaining how indirect reciprocity could evolve has therefore remained problematic. In all previous treatments of indirect reciprocity, individuals are assigned potential recipients and decide whether to cooperate or defect based on their reputation. A second way of achieving discrimination is through partner choice, which should enable individuals to avoid defectors. Here, I develop a model in which individuals choose to donate to anyone within their group, or to none. Whereas image scoring with random pairing produces cycles of cooperation and defection, with partner choice there is almost maximal cooperation. In contrast to image scoring with random pairing, partner choice results in almost perfect contingency, producing the correlation between giving and receiving required for cooperation. In this way, partner choice facilitates much higher and more stable levels of cooperation through image scoring than previously reported and provides a simple mechanism through which systems of helping those who help others can work.

## Introduction

If an individual helps someone on the basis that they have helped others, indirect reciprocity can be said to be in action [1]. Indirect reciprocity is an important concept because it is integral to the notion that we may benefit from being seen to be cooperative. Furthermore, it is not dependent upon repeated meeting between the same pair of individuals [2]. These factors suggest that indirect reciprocity may have been important in the development of human sociality, and may have co-evolved with morality and language [3].

Models of indirect reciprocity show that cooperation (*sensu* paying a cost to benefit another, e.g. [4]) is possible, but that it is cyclically unstable, with defection being as likely as cooperation [2]. Image scoring, which represents cooperating and defecting histories positively and negatively, has an Achilles heel because withholding help from defectors harms ego’s reputation, yet providing help erodes the discrimination required for cooperation. Experiments show that people do reward those who are generous to others [5–8] and that this may contribute to solving social dilemmas [9,10]. Thus, despite image scoring being unstable in theory, a tendency to base behaviour on reputations is evident in practice. One possibility is that people use the ‘standing’ [11] construct, which differs in that individuals can justifiably defect on a defector without their reputations being harmed [12]. Such a strategy resolves the issues with evolutionary stability [13] but the requirement for second order reputational assessment increases the information gathering and processing demands, restricting its use in practice [5]. There therefore remain fundamental difficulties in understanding both theoretically how indirect reciprocity might have evolved [14], and empirically why people give more to co-operators.

There are two ways in which discrimination of cooperators may be effected. In all previous treatments of indirect reciprocity, individuals have been presented with potential recipients and have had to decide how they would play, contingent upon their own strategy and the partner’s reputation [3]. This question of how to respond to a partner’s previous moves has been referred to as ‘partner control’ [15] and in the context of direct reciprocity, strategies related to Tit-for-Tat [16] have received considerable attention. The second way to achieve discrimination is through partner choice. This is an important driver of social behaviour and a powerful means by which direct reciprocity can be stabilized [17–20]. Here I introduce for the first time the possibility of partner choice (strictly speaking recipient choice since interactions are one-off and unidirectional) into a model of indirect reciprocity (see ref. [21] re the importance of making this advance). I consider social groups in which individuals know the reputations of others and can choose to whom to donate. My hypothesis is that provided there is at least one other cooperator in a group, then individuals shouldn’t need to interact with defectors at all, so a key issue with image scoring can be avoided and indirect reciprocity should lead to cooperation at a stable, high level (see ref. [22] for similar reasoning and refs. [19,23–25] for examples of the importance of opt-outs).

## Methods

I modelled indirect reciprocity using a form of first-order reputation indexing related to image scoring [2]. Scores were initialised at 0 for each individual in each generation (as in [2,13]), reflecting the fact that individuals begin their lives without experience. Scores then became -1 following defection and 1 following cooperation [26]. Strategies were defined by their thresholds *k*, where *k* = -1 denotes individuals that cooperated unconditionally; *k* = 0 strategists cooperated provided their partner had never played or had cooperated; *k* = 1 strategists cooperated provided the partner had cooperated; and *k* = 2 strategists never cooperated. Individuals interacted in groups of size *g* with default *g* = 100.

Interactions proceeded by the program randomly choosing a focal individual (i.e. an individual upon whom operations are performed by the program) which then had an opportunity to donate to a partner from the same group, conditional upon its own strategy and the partner’s image score. Depending on the focal’s strategy, partners were either assigned randomly or were selected as having positive image scores. Partner choice was implemented by presenting individuals with a randomly chosen partner from their group, and allowing the focal to reject the partner until either (1) the partner had a positive image score, in which case partner choice was successful; or (2) the program reached a giving up time that was set so that approximately all group members were sampled in the search for one with a positive image score. In this case, the focal had to settle for the last partner randomly assigned by the program [19].

In treatment conditions with random partner assignment, focals were assigned partners at random from their group by the program. Individuals with *k* = 2 always had partners assigned at random: *k* = 2 individuals always defect, so there is no question of choosing who to give to, as they never give. They are therefore simply assigned partners at random. There are no consequences for the randomly assigned individual: they don’t receive anything, nor do they take any action. The only consequence is that the *k* = 2 individual gains a negative image score, which would be the same result regardless of the partner.

This interaction process was repeated such that every group member had on average *m* meetings with every other, either as donor or recipient, where by default *m* = 1. If the focal donated, it incurred cost *c* = -1 and the partner received benefit *b* = 2. The donation cost-benefit ratio was set to 1:2 rather than the 1:10 employed in some earlier studies [2] in order to be conservative regarding the evolution of cooperation. To avoid negative payoffs (which should not occur in reality provided cooperating costs a small proportion of available resources), all focals had their payoffs incremented by 1 each time they played.

A meta-population structure was used in which *p* = 10,000 individuals were distributed evenly over *i = 100* islands, where islands are units in which reproduction is structured. Note that the default island size was equal to one group, but the parameters were distinct so that interaction group size could be varied without affecting reproduction and drift. Simulations started with equal proportions of all strategies and neutral image scores. This practice follows previous authors e.g. [2], but it can be noted that the results reported were effectively identical when starting from a population of all unconditional defectors. Simulations were evolutionary in that those strategies accumulating the highest payoff produced most offspring. Reproduction was based on the success of a strategy both within and between islands. The payoffs of all strategies were summed, then individuals were assigned strategies in the next discrete generation in proportion to the relative payoff of each strategy. An individual for the next generation was derived locally with probability 0.9 and globally with probability 0.1. This reduced the potential for genetic drift and allowed migration of successful strategies to other islands [13,26]. Population size was therefore kept constant, with each generation being fully replaced by the next. Reproduction was accompanied by mutation: with probability μ = 0.01 an individual’s strategy was replaced at random (any strategy could change to any other strategy without having to track through an imposed sequence). Simulations were run for 10,000 generations.

Phenotypic defectors are individuals carrying a cooperative strategy yet defecting (which is postulated to occur due to e.g. lack of resources) [27,28]. Phenotypic defectors were included in selected simulations in order to allow comparison with earlier work that has shown they may play a role in stabilizing cooperation. In simulations with phenotypic defectors, these were introduced by constraining 10% of agents in each generation from cooperating, regardless of their genetic strategy. The payoffs of those assigned to be phenotypic defectors within any one generation nevertheless contributed to the relative payoff of the genetic strategy they carried.

In the model, as in most models of cooperation, image scores depend only upon the last move. In a few models of indirect reciprocity (e.g. [13]), strategy thresholds are constant for each player across rounds yet image scores are cumulative. This results in a situation whereby it can pay to attend to one’s own image score and cooperate when in poor standing but not when in good standing as there may be little to be gained because one’s image score already exceeds the population threshold for cooperating. The model presented here avoids such unrealistic internal inconsistencies and thereby avoids the artefactual complexities reported in [13].

## Results

### Evolution of cooperative strategies

**Fig 1:**
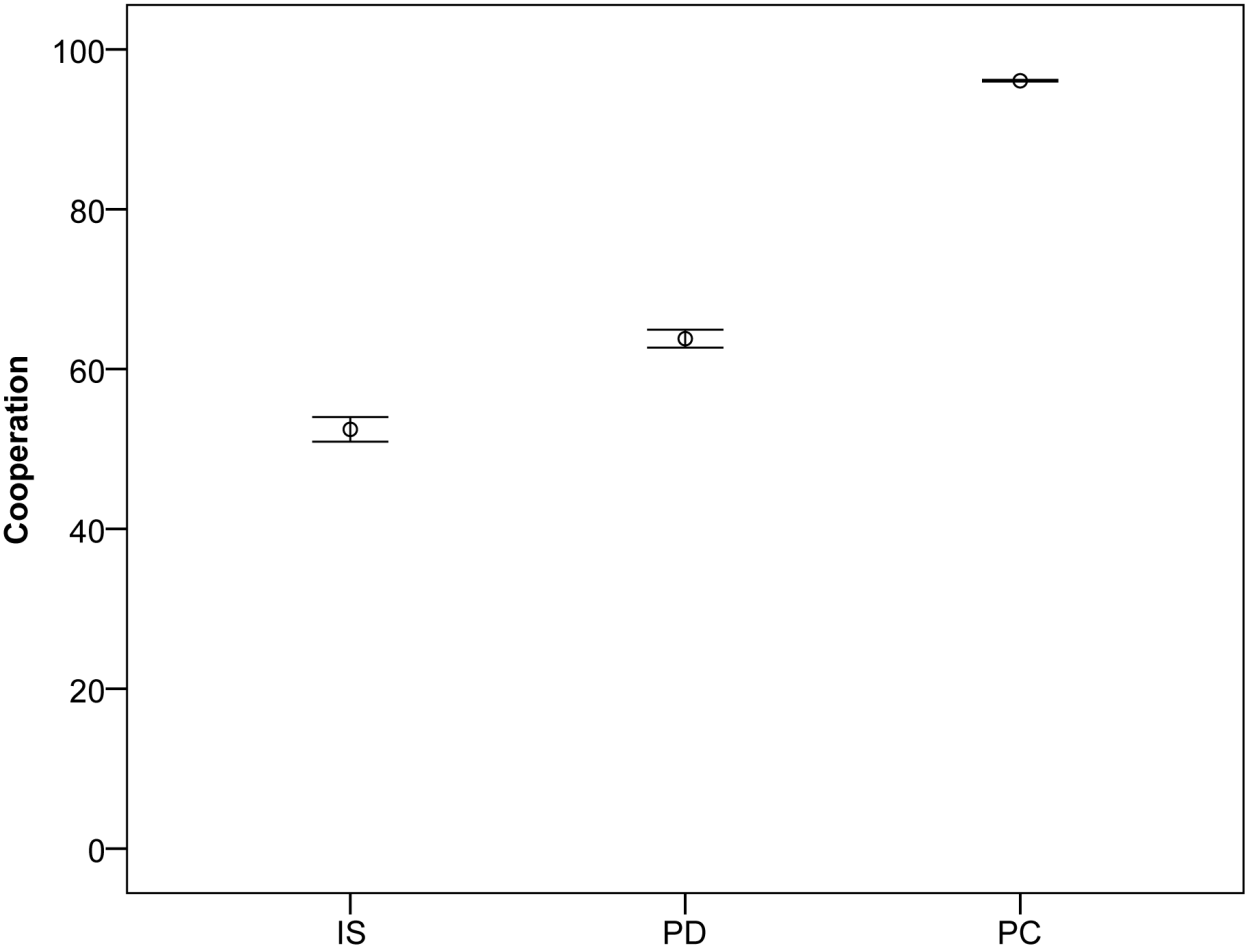
Cooperation with image scoring and partner choice. Percentage cooperation in simulations with pure image scoring with random assignment (IS); incorporating phenotypic defectors (PD); and with partner choice (PC). Cooperation is plotted as the mean ± SEM across 10 simulations of within simulation means each calculated across all 10,000 generations. One way ANOVA, F_2,27_ = 422.42; p<0.0005; post-hoc Scheffe comparisons all significant at 0.0005 level.

I first replicated results for ‘pure’ image scoring with random assignment of partners and with the inclusion of ‘phenotypic defectors’—individuals that carry a genetic basis for cooperation yet only have the capacity to defect [27,28]. Looking first at the levels of cooperation which emerge (Fig 1), the inclusion of phenotypic defectors does lead to an increase [26]. However, introducing partner choice (without phenotypic defectors) produces a much greater effect, almost doubling cooperation from 52.46 ± 1.54 with random assignment to 96.07 ± 0.11 with choosiness (unless otherwise stated, means ± SEM are calculated across 10 simulations, where within simulation means are calculated over 10,000 generations with the default parameters). Thus, although introducing phenotypic defectors has some appeal in theory, partner choice is much more effective in increasing cooperation.

**Fig 2:**
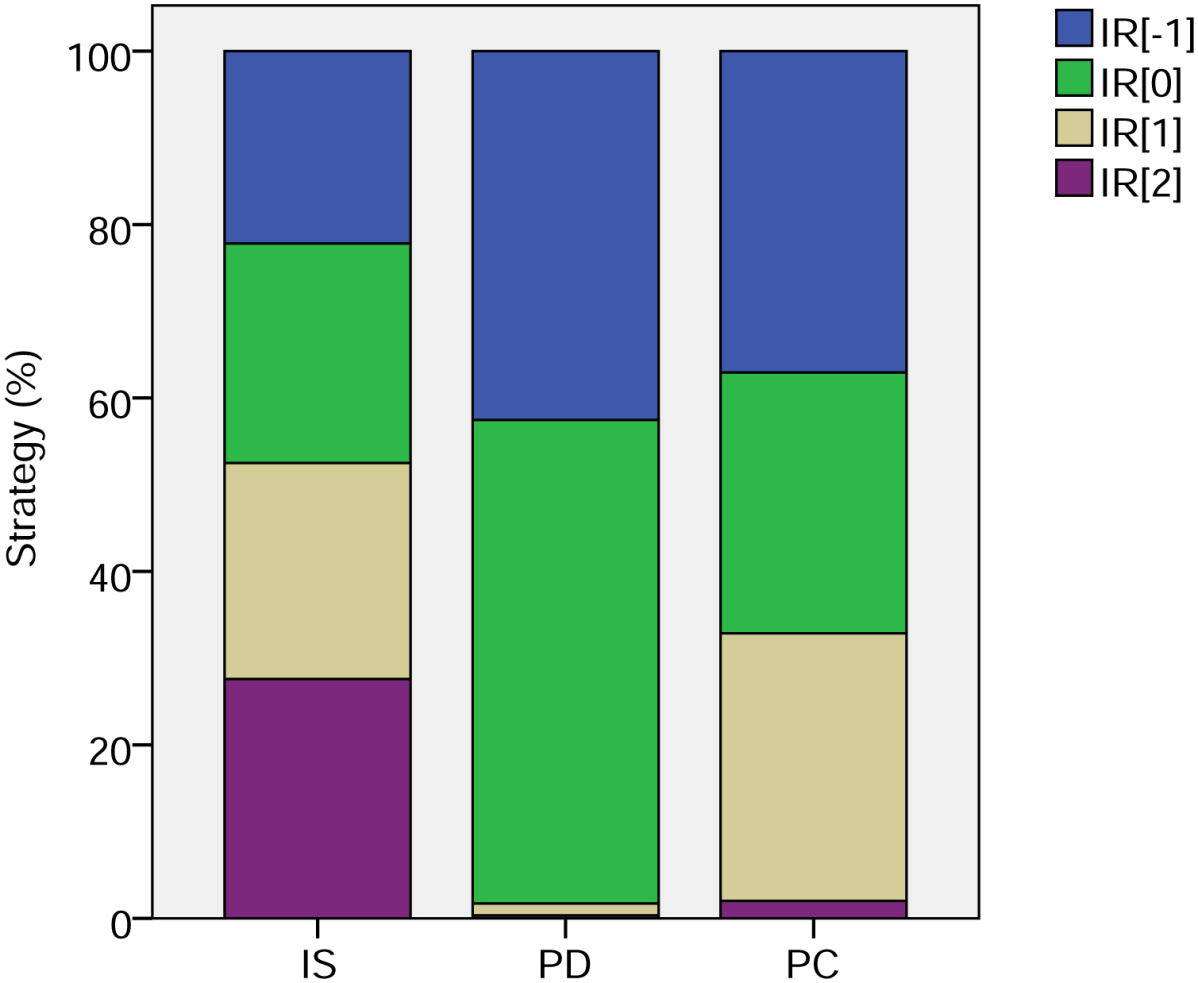
Strategies of image scoring and partner choice. Strategy mixes with pure image scoring with random assignment (IS); incorporating phenotypic defectors (PD); and with partner choice (PC). Strategies are labelled as IR[*k*] where *k* is the strategy threshold. Thus *k* = -1 denoted individuals that cooperated unconditionally; *k* = 0 strategists cooperated provided their partner had never played or had cooperated; *k* = 1 strategists cooperated provided the partner had cooperated; and *k* = 2 strategists never cooperated. Proportions are shown as means over 10 simulations of within simulation means calculated across 10,000 generations.

Examining the proportions of each strategy (Fig 2) reveals that with pure image scoring there is an almost even mix of all strategies. With phenotypic defectors, there is a balance between unconditionally cooperative strategies and those which cooperate with those with at least neutral image scores. With partner choice, again almost all strategies are cooperative, these now being divided between the cooperative thresholds (i.e. all except unconditional defectors). This reflects the fact that all can now discriminate through choice of partner, so the threshold becomes less important. So both processes lead to the dominance of potentially cooperative strategies, though in practice, the presence of phenotypic defectors constrains the number of actual cooperative acts (as reported in the discussion of Fig 1).

**Fig 3:**
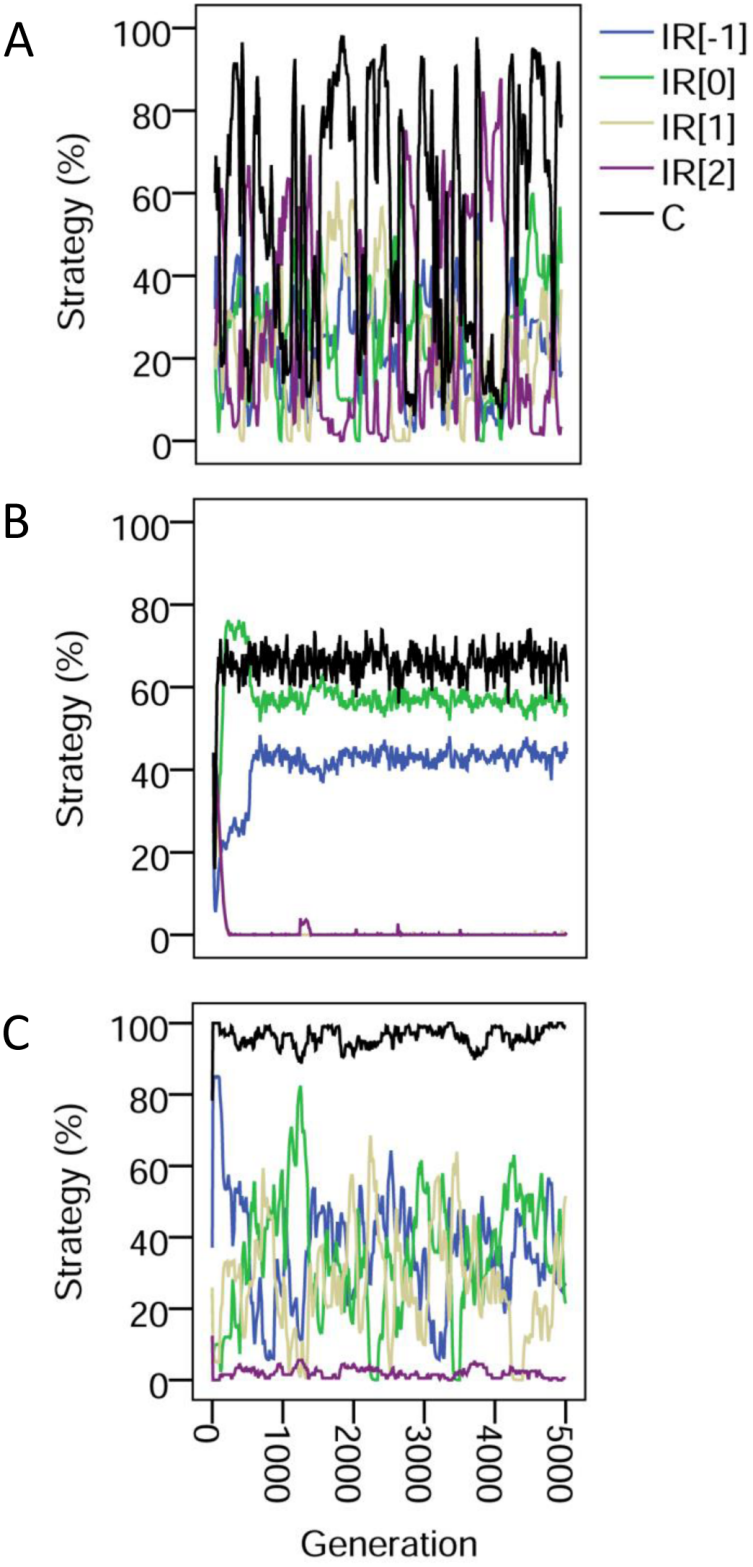
Dynamics of image scoring and partner choice. The evolutionary dynamics of indirect reciprocity strategies for (A) pure image scoring with random assignment; (B) with phenotypic defectors; (C) with partner choice. The first of 10 simulations is shown in each case. Strategies are labelled as IR[*k*] where k is the strategy threshold. Thus *k* = -1 denoted individuals that cooperated unconditionally; *k* = 0 strategists cooperated provided their partner had never played or had cooperated; *k* = 1 strategists cooperated provided the partner had cooperated; and *k* = 2 strategists never cooperated. C is cooperation, plotted as the proportion of cooperative moves.

Examining the evolutionary dynamics further helps us to understand this mix of strategies (Fig 3). With pure image scoring, the dynamics of all strategies are highly unstable with cycles of cooperation and defection. These cycles are stabilized by the addition of phenotypic defectors, however cooperation asymptotes at a moderately high level. In contrast, partner choice allows total cooperation to reach a stable, almost maximal level. Note that the strategy thresholds actually wander below the always-defect level: once a rule of choosing a partner with a positive image score becomes established then the strategy threshold is released from selection (provided it is below the unconditional defection level).

The simulations reported here were initialized with an even mix of strategies, however initializing with 100% unconditional defectors results in indistinguishable average levels of cooperation and frequencies of strategies, confirming that partner choice can allow cooperation to invade.

### Discrimination

**Fig 4:**
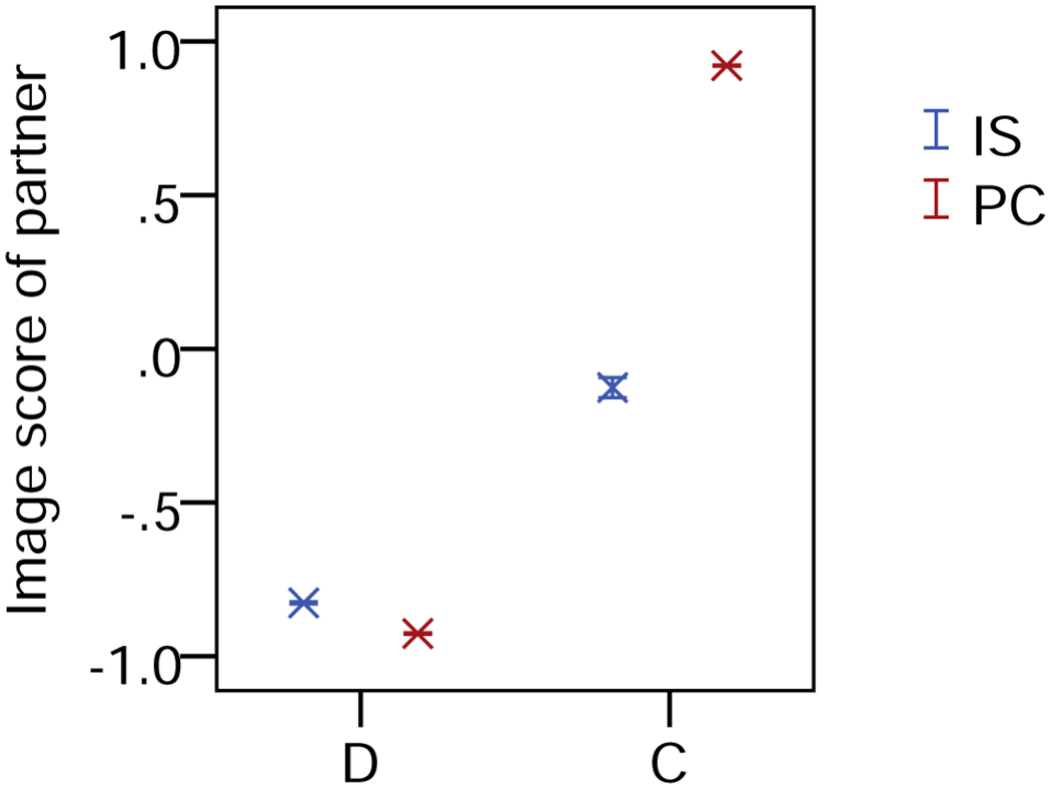
Discrimination in image scoring and partner choice. Discrimination between recipients as shown by the image scores of those with whom focal individuals cooperated (C) or defected (D), plotted as means ± SEM for pure image scoring with random assignment (IS) and partner choice (PC).

Partner choice is clearly effective at increasing cooperation via indirect reciprocity, but can we show that individuals are expressing a partner preference? The mean image score of those defected upon (on the scale from -1 to 1) was -0.83 ± 0.00 with pure image scoring versus -0.93 ± 0.00 with partner choice (the difference from -1 reflecting defection when no individual with positive image score was available; Fig 4), so choice did bring some degree of increased selection against defectors (*t*_18_ = 25.66, *p*<0.0005). A more marked difference is apparent when looking at those donated to. Here the mean image score was -0.13 ± 0.03 under partner assignment but 0.92 ± 0.00 under partner choice (the difference from 1 reflecting the fact that where recipients with positive image score are unavailable, focals will cooperate according to their threshold; *t*_18_ = -31.87, *p*<0.0005). Thus, with pure image scoring there is on average close to no discrimination by image score of who is given to, while under partner choice, there is almost pure preference for co-operators.

### Evolution of choice

I have shown that choosiness does support cooperation through indirect reciprocity, but would a strategy of being choosy invade a non-choosy population? I ran simulations in which each individual’s strategy was a combination of two independent elements: an indirect reciprocity threshold determining whether individuals would donate to partners (the thresholds being as described in the Methods), and a choosiness element which determined whether the individual would accept a random partner as a recipient or would express a preference for individuals with positive image score (the implementation of the partner choice routine being as previously described in the Methods). The result was that partner choice and random pairing were both maintained in the population: those individuals using partner choice accounted for approximately half (49.73 +/- 0.76%) of strategies. A high level of cooperation was found, with occasional invasion by defection lowering the mean to 87.68 ± 0.74%. Cooperation was associated with the spread of those choosy strategies that are potentially cooperative (the sum of the frequencies of strategies with *k*<2) correlated with cooperation (*r* = 0.71, *p* = 0.021, *n* = 10 simulations); whereas the potentially cooperative non-choosy strategies did not (*r* = 0.02, *p* = 0.95, *n* = 10).

### Direct reciprocity

When modelling indirect reciprocity it is instructive to limit the possibility for direct reciprocity so that we can be sure any reciprocation is from third parties. The simplest way to do this is to restrict the number of interactions so that the probability of any agent having the opportunity to receive back from an agent it has donated to is too small to determine the outcome [2]. In the simulations presented, every agent had on average one interaction either as donor or recipient with every other individual (*m* = 1). To determine the effect of reducing the possibility of direct reciprocation still further, I repeated the simulations with 1/50 the number of dyads (*m* = 0.02), so each agent had on average approximately only one interaction as donor and one as recipient in total in each generation. Mean cooperation over the 10,000 generations was 95.95 ± 0.14%, so the result is unchanged from the 96.07 ± 0.11 found under the default conditions of *m* = 1. We can therefore conclude that direct reciprocation is not a factor in explaining the high levels of cooperation found with indirect reciprocity and partner choice.

### Phenotypic defectors and partner choice

I have considered the introduction of phenotypic defectors and of partner choice separately. For completeness, I also investigated introducing both simultaneously. This actually lowers cooperation from the level of 96.07 ± 0.11 with partner choice and default parameters to 81.36 ± 0.00. This can be understood as being an inevitable consequence of introducing individuals that do not cooperate into an almost fully cooperative environment and so is not explored further here.

### Group size

**Fig 5:**
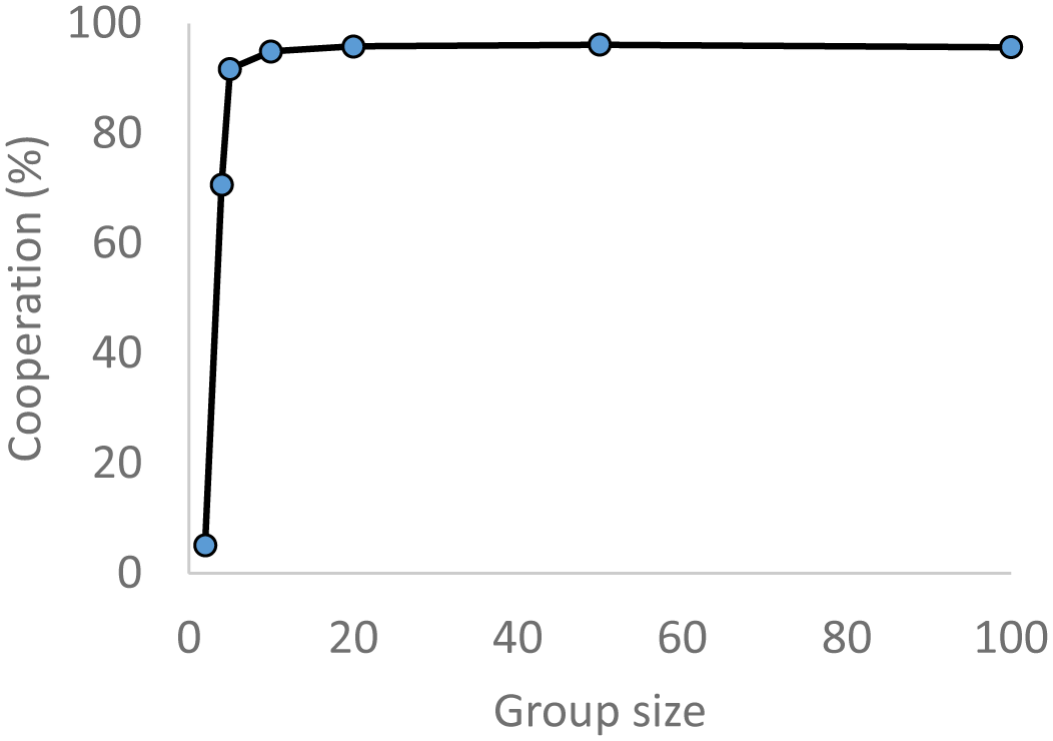
Cooperation and group size. The mean level of cooperation across 10 simulations, each of 10,000 generations is shown in relation to the group size in which individuals interacted using image scoring with partner choice, maintaining *m* = 1 and island size = 100. Standard errors are too small to be visible.

As group size decreases, there may be less opportunity to find a co-operator from within the group and so it may be harder for cooperation to get established. I therefore investigated reducing the group size from the default of 100 to 10 while retaining *m* = 1. Results were very similar, with a marginal reduction from 96.07 ± 0.11 to 94.91± 0.18% cooperation (Fig 5). Even with a group size as low as 4, cooperation remains at a high level of 70.66 ± 1.91%. This reduced mean at the lowest group sizes results from cycles of invading defection, as happens without partner choice. This is consistent with the fact that at these group sizes there is effectively very little choice available. Very small group sizes are not of interest for indirect reciprocity since the point is to explain cooperation in large groups with few meetings per individual. In groups of 2, maintaining the parameter *m* = 1 to eliminate potential for direct reciprocity means that each is on average a donor or recipient only once so reciprocity cannot produce a profit.

### Standing

The reputation assignment mechanism of standing [11,13] provides an alternative to image scoring, with the benefit of greater evolutionary stability but the drawback of requiring second order information on prior behaviour in order to distinguish those defections which are ‘justifiable’ because they are with others who have themselves defected [12]. A version of the standing mechanism was implemented as in [26] by modifying the reputation scoring routine such that defection only produced a bad reputation when it was with an agent that had previously either cooperated or not moved, otherwise the focal agent’s reputation remained unchanged. Based on previous results showing that standing can engender high levels of cooperation under indirect reciprocity [26], I predicted that partner choice would have little additional impact on the evolutionary dynamics. This was upheld, with standing in a partner assignment regime giving 84.71 ± 0.47% cooperation (with default parameters) and partner choice increasing this to 96.22 ± 0.09%, a figure almost identical to that with image scoring and partner choice (96.07 ± 0.11). Thus, when individuals can choose partners, the benefits of having second order information disappear: partner choice can produce very high levels of cooperation even without the complexity of knowing the context in which players gained their reputations.

### Costs of choice

I have assumed that individuals in groups have information on their group-mates and can select with whom they interact in a cost-free way. However, it is also possible that if potential partners are presented one at a time, there may be some cost in rejecting one and holding out for a better partner. This may take the form of an opportunity cost such that with a given probability, the focal then fails to find a further partner. This was implemented such that a focal could only proceed with the interaction with probability *p* raised to the power of the number of partners it had rejected before finding one that was acceptable. If this condition was not satisfied then a new focal was chosen. This procedure meant that the total number of interactions was retained, but that non-choosy individuals had a greater proportion of the interactions. I found that, as one might predict, costs of choice did reduce both cooperation and choosinesss, but that both were remarkably robust. With *p* = 0.5 (i.e. each time a partner was rejected it halved the chance of that focal being able to play) cooperation was retained at 80.38 ± 1.26% with 42.98 ± 0.86% of individuals being choosy. Even with *p* = 0.1, cooperation was 72.34 ± 1.31% and choosy strategies accounted for 36.61 ± 0.88%. Thus the cost of choice is an important factor to consider but it does little to impede the evolution of cooperation through partner choice.

## Discussion

To conclude, partner choice overcomes the issues that image scoring has with instability, and allows reliably high levels of cooperation without direct reciprocation even in the smallest groups. This is achieved through a simple mechanism without invoking the complexities of phenotypic defectors [27,28], standing [11,13], tolerance [29], or evolving social networks [14]. The key to the effectiveness of choice is that it allows much higher levels of discrimination. Under pure image scoring, those donated to are as likely to be defectors as co-operators. Cooperation requires a correlation between giving and receiving, and partner choice assures this, allowing almost perfect contingency such that only those who have given are given to. Unlike under pure image scoring there is no temptation to cooperate with defectors just to preserve one’s own reputation—instead they can simply be avoided.

The impact of partner choice can be understood within the existing analytical framework for understanding image scoring. If cooperation depends upon the level of “acquaintanceship” or *q* [30] then rather than being limited by the average of *q* across all those an individual might meet, individuals can choose to interact with those for which their knowledge of cooperativeness is maximal. Thus the condition for cooperation to work under indirect reciprocity is much more readily satisfied.

The model presented here should be tested using an experiment in which people are able to choose who (if any) in their group to donate to. The prediction is that people should demonstrate strong discrimination between recipients, resulting in a high level of cooperation through indirect reciprocity. One study of cleaner fish presented individuals with a choice of cooperating and defecting partners and did find evidence of image scoring [31].

The results here provide further support for the role of partner choice in driving reputation-based cooperation. A number of studies have shown how those individuals who are seen to be the most cooperative are the most sought after partners for alliances, or repeated mutually-beneficial relationships, and that this can drive strategic investment in cooperation [32,33]. This study provides evidence that partner choice can also stabilize cooperation in the context of one-off, one-way donations without re-meeting.
